# The role of the unusual threonine string in the conversion of prion protein

**DOI:** 10.1038/srep38877

**Published:** 2016-12-16

**Authors:** Romany Abskharon, Fei Wang, Kayla J. Vander Stel, Kumar Sinniah, Jiyan Ma

**Affiliations:** 1 Center for Neurodegenerative Science, Van Andel Research Institute, Grand Rapids, MI 49503, USA; 2 National Institute of Oceanography and Fisheries (NIOF), 11516 Cairo, Egypt; 3 Department of Chemistry and Biochemistry, Calvin College, Grand Rapids, MI 49546, USA

## Abstract

The conversion of normal prion protein (PrP) into pathogenic PrP conformers is central to prion disease, but the mechanism remains unclear. The α-helix 2 of PrP contains a string of four threonines, which is unusual due to the high propensity of threonine to form β-sheets. This structural feature was proposed as the basis for initiating PrP conversion, but experimental results have been conflicting. We studied the role of the threonine string on PrP conversion by analyzing mouse *Prnp*^*a*^ and *Prnp*^*b*^ polymorphism that contains a polymorphic residue at the beginning of the threonine string, and PrP mutants in which threonine 191 was replaced by valine, alanine, or proline. The PMCA (protein misfolding cyclic amplification) assay was able to recapitulate the *in vivo* transmission barrier between PrP^a^ and PrP^b^. Relative to PMCA, the amyloid fibril growth assay is less restrictive, but it did reflect certain properties of *in vivo* prion transmission. Our results suggest a plausible theory explaining the apparently contradictory results in the role of the threonine string in PrP conversion and provide novel insights into the complicated relationship among PrP stability, seeded conformational change, and prion structure, which is critical for understanding the molecular basis of prion infectivity.

Transmissible spongiform encephalopathies (TSEs), or prion diseases, are a group of fatal neurodegenerative diseases that includes Creutzfeldt-Jakob disease (CJD) in humans, scrapie in sheep and goats, bovine spongiform encephalopathy (BSE) in cattle, and chronic wasting disease (CWD) in cervids^1^. The prion hypothesis posits that the conversion of normal host-encoded prion protein (PrP) is central to the pathogenesis of these diseases^2^,^3^. The normal PrP isoform (PrP^C^) is a cell surface glycoprotein that is soluble, protease-sensitive, and rich in α-helices. During the disease, some PrPs convert to the aberrantly folded isoform (PrP^Sc^), which is aggregated, resistant to protease digestion, and rich in β-sheets^3^,^4^,^5^. The pathogenic PrP^Sc^ is able to seed a conformational change of normal PrP^C^ into PrP^Sc^, resulting in PrP^Sc^ accumulation and neurodegeneration. Decades of research have provided compelling evidence supporting the hypothesis^6^,^7^.

The normal isoform, PrP^C^, contains a long unfolded N-terminal region and a C-terminal globular domain consisting of three α-helices and two short anti-parallel β-strands^8^,^9^,^10^,^11^. Although small changes in the amino acid sequence do not significantly alter the overall structure of PrP^C^^9^,^10^, they can severely alter prion transmissibility^12^,^13^,^14^,^15^,^16^. PrP sequence variations in different species create strong barriers against inter-species prion transmission^17^,^18^. Within the same species, PrP polymorphisms also greatly influence the susceptibility to intra-species prion transmission^12^,^15^,^19^,^20^. A classic example of an intra-species barrier is the mouse PrP polymorphism of allele a (*Prnp*^*a*^; Leu at residue 108 and Thr at residue 189) and allele b (*Prnp*^*b*^; Phe at residue 108 and Val at residue 189), which drastically alter the length of the incubation period of prion disease^12^,^20^,^21^. Elegant studies with knock-in mice revealed that the most efficient prion transmission requires both residues in the host PrP to match those of the inoculum PrP^20^. While both residues 108 and 189 affect the incubation time, residue 189 plays a major role in determining the efficiency of prion transmission^20^.

Residue 189 is within the highly conserved string of 4 threonines located at the C-terminus of α-helix 2 of PrP (Fig. 1)^22^. Because threonine tends to form β-sheet structures^23^, such a sequence of β-sheet-prone threonines in a helix is highly unusual. This local instability has been proposed as the underlying structural basis for initiating the PrP^C^-to-PrP^Sc^ conversion^24^. Consistent with this hypothesis, the C-terminus of α-helix 2 has been found to be unstable^25^,^26^, and the peptide corresponding to helix 2 is able to form amyloid fibrils that can act as seeds for full-length wild-type PrP^27^. Replacing threonines with α-helix-prone alanines stabilizes the PrP^C^ structure and prevents *in vitro* PrP aggregation^28^. A thermodynamic study of the α-helix 2 peptide showed that the free energy separating the α-helix and β-sheet conformations of this peptide is very small, indicating that the structural behavior of this peptide is likely determined by the environment^29^. Indeed, compounds binding to this region have been shown to prevent PrP conversion and increase the survival of diseased animals^30^,^31^,^32^.

Interestingly, the insertion of extra amino acids in the C-terminus of α-helix 2 does not affect the ability of mutant ovine PrPs to convert to the infectious PrP^Sc^ conformation^22^. Further, a comprehensive mutagenesis study of mouse PrP showed that individually replacing the amino acids in the C-terminus of α-helix 2 with any other amino acid does not significantly influence the formation of infectious prions in scrapie-infected N2a cells^33^. Very recently, Munoz-Montestino reported that deleting 4 or 5 amino acids in this region (including all four threonines) does not affect the conversion of ovine PrP to infectious prions in RK13 cells^34^. These data apparently contradict the observations from prion transmission studies in *Prnp*^*b*^knock-in mice, which showed that the threonine 189 (mouse numbering) significantly affects disease incubation times^20^.

The above studies were performed on cultured cells or living animals, and multiple factors might alter the efficiency of PrP conversion in these complicated systems (such as cell metabolic state, PrP presentation on the cell surface, the presence of multiple cofactors, etc.). Therefore, we chose two relatively simple *in vitro* assays that recapitulate many characteristics of PrP^C^-to-PrP^Sc^ conversion to study the influence of the threonine string in prion conversion. The first is protein misfolding cyclic amplification (PMCA), which consists of successive sonication and incubation steps^35^. We previously showed that this technique can convert bacterially expressed recombinant PrP (recPrP) into aggregated and proteinase K (PK)-resistant conformer in the presence of cofactors^36^,^37^. This conformational change produces recPrion, the pathogenic conformer of recPrP, that causes bona fide prion disease in wild-type mice with pathological properties identical to those of naturally occurring prions^36^,^38^. In addition to PMCA, we also used a denaturant-induced recPrP amyloid fibril formation assay^39^,^40^,^41^, which is commonly used to model PrP conversion.

Using these two assays, we compared recombinant PrP^a^ and PrP^b^, two derivatives of PrP^a^ bearing either Phe at residue 108 (108 F) or Val at residue 189 (189 V), and three PrP^a^ mutants that replace Thr 191 with either valine, alanine, or proline. Our results offer new insights into the conversion of PrP into an infectious prion.

## Results

### PrP^b^ polymorphism compromises recPrP conversion by PMCA

The presence of Phe 108 and Val 189 in PrP^b^ creates a strong barrier to the transmission of PrP^a^-based prion strains^20^,^42^. The recPrion was generated *de novo* by PMCA from recPrP^a^ protein^36^ and it has been propagated with recPrP^a^ for years. To determine whether there is a transmission barrier between recPrion and recPrP^b^, we propagated recPrion with recPrP^b^ or with the PrP^a^-derived mutants recPrP^L108F^ and recPrP^T189V^. PMCA was carried out as described previously^38^,^43^; the PMCA substrate consisted of recPrP plus two cofactors, synthetic POPG and total RNA isolated from normal mouse liver^38^,^43^. For each recPrP variant, PMCA was carried out for six rounds, and the generation of the C-terminal PK-resistant PrP band at round 6 was considered a successful propagation. As a positive control, recPrP^a^ was successfully propagated (Fig. 2 and Table 1). In contrast, PrP^b^ and the two derivative mutants failed to propagate in all 20 trials (Fig. 2 and Table 1). These data suggested that, similar to the barrier observed in animal study^20^, there is a barrier for propagating recPrP^a^-adapted recPrion to recPrP^b^ and that both polymorphic residues 108 and 189 contribute to the barrier.

### PrP^b^ polymorphism at residues 108 and 189 differently affect recPrP amyloid fibril growth

Next, we analyzed the influence of PrP^b^ polymorphism on PrP conversion with the less stringent but commonly used *in vitro* recPrP amyloid fibril growth assay^39^. In unseeded reactions, the amyloid fibril formation by recPrP was highly variable (Fig. 3a). Within the 96-h experiment, 4 out of 5 recPrP^a^ replicates formed amyloid fibrils *de novo* after a lag time of about 56 h. For recPrP^b^, only 1 out of 5 replicates formed amyloid fibrils *de novo* after a lag time of about 79 h, and none of the other 4 recPrP^b^ replicates formed fibrils within the 96-h experiment (Fig. 3a). These results were consistent with a previous report showing that recPrP^b^ has a reduced capability to form amyloid fibrils^44^.

Surprisingly, when the two PrP^a^-derived mutants recPrP^L108F^ and recPrP^T189V^ were tested, they behaved differently. Within the 96-h experiment, 4 out of 5 recPrP^L108F^ replicates formed amyloid fibrils *de novo* with a lag time similar to that of recPrP^a^ (Fig. 3a). The recPrP^T189V^ however, behaved similar to recPrP^b^ (Fig. 3a), that is, only 2 out 5 recPrP^T189V^ replicates formed amyloid fibril *de novo* within 96 h, and the average lag phase was around 91 h. The same trend was observed in seeded reactions, in which all four recPrPs were seeded with the same recPrP^a^ fibril seed and amyloid fibril growth was detected in all samples with less variation. The recPrP^L108F^ behaved like recPrP^a^, both showing a rapid growth of amyloid fibrils (Fig. 3b). In contrast, recPrP^T189V^ and recPrP^b^ amyloid fibril growth had a longer lag phase, and the intensity of ThT fluorescence signal was much lower (Fig. 3b). Since these reactions were monitored at the same time, in the same plate, and under identical condition, the difference in amyloid fibril growth is likely resulted from the different amino acid sequences of recPrP.

Overall, these results supported the idea that the recPrP^b^ protein has a reduced capability to form amyloid fibrils, which is largely due to the influence of residue 189 of PrP. These findings are consistent with the observations in animal studies that residue 189 is the major determinant for the transmission barrier between PrP^a^ and PrP^b^. However, the amyloid fibril growth results regarding residue 108 were different from *in vivo* findings^20^.

### The influence of mutations at residue 191 on recPrP conversion

Because residue 189 has a larger influence on the PrP^a^/PrP^b^ transmission barrier and it is at the beginning of the 4-threonine string of α-helix 2, it is reasonable to ask if changing another threonine residue within that string would have similar effects. Thus, we mutated Thr-191 in recPrP^a^ to Val to mimic the recPrP^T189V^ change in recPrP^b^. We also mutated Thr-191 to α-helix-prone Ala or to the secondary structure breaker Pro. All three mutants were expressed, purified, and subject to PMCA.

Unlike recPrP^T189V^, recPrP^T191V^ was able to propagate recPrion by PMCA, but the efficiency was lower (about 60%) and the PK-resistant band at the 6^th^ round was generally weaker than that of recPrP^a^ (Fig. 4 and Table 1). The recPrP^T191A^ mutant had an even lower efficiency, only around 25% of reactions completed six rounds of propagation, and the PK-resistant band was also weaker than that of recPrP^a^ (Fig. 4 and Table 1). On replacing Thr 191 with Pro, none of the 20 trials yielded successful propagation (Fig. 4 and Table 1). Thus, relative to Thr-189, Thr-191 is more tolerant to amino acid replacement (with the exception of Pro): both Val and Ala at this position can be tolerated but with less-efficient recPrion propagation by PMCA.

All residue-191 mutants were assayed for amyloid fibril growth. In unseeded reactions, 4 our 5 recPrP^T191A^ or recPrP^T191V^ were able to form fibrils *de novo*, which were similar to wild-type recPrP^a^ (4 out 5 replicates formed amyloid fibrils) except for the longer lag phases (Fig. 5a). For recPrP^T191P^ however, only 1 out of 5 replicates formed fibril *de novo* within the 96-h experimental period, and the lag phase was 94 h (Fig. 5a). When fibril growth was seeded with recPrP^a^ fibril seed, all three residue-191 variants grew amyloid fibrils robustly (Fig. 5b). Thus, except for the reduced ability of recPrP^T191P^ to form amyloid fibrils *de novo*, no significant difference in amyloid fibril growth was found when Thr-191 was replaced with Val, Ala, or Pro.

### Detecting prion infectivity of converted recPrP variants using cell infection assay

The Elispot plate–based cell infection assay is a sensitive and quantitative prion infectivity measure^45^,^46^. We used CAD5 cells, a clonally selected cell line that is susceptible to infection by most mouse prion strains^45^,^46^, to test PMCA products for two purposes. First, we wanted to determine whether the *in vitro* converted recPrP variants were infective. Second, because the endogenous PrP in CAD5 cells is wild-type PrP^a^, using converted recPrP variants to infect the cells would allow us to evaluate the effect of amino acid mismatch on PrP conversion.

The round-6 PMCA products were used for the assay. As shown in Fig. 6, no infectivity was detected in the recPrP variants that did not produce a detectable C-terminal PK-resistant band, i.e., recPrP^b^, recPrP^L108F^, recPrP^T189V^, and recPrP^T191P^ (Fig. 6). This result supports the notion that PrP conformation is the main determinant of prion infectivity. The three recPrP variants that generated the PK-resistant conformer at round 6 all had prion infectivity (Fig. 6). Although the PK-resistant bands of recPrP^T191V^ and recPrP^T191A^ were much weaker than that of recPrP^a^ (Figs 2 and 4), the infectivity of recPrP^T191V^ was comparable to that of wild-type recPrP^a^ and the infectivity of T191A mutant was lower by about one order of magnitude. Nonetheless, the undiluted samples of recPrP^a^, recPrP^T191V^, and recPrP^T191A^ produced similar numbers of cells carrying PK-resistant PrP^Sc^ (Fig. 6), suggesting that the amino acid mismatch at 191 did not significantly affect the seeded conversion process.

### Thermal stability of recPrP variants

The sequence of four threonines at the C-terminus of α-helix 2 is unstable, which has led to it being proposed as a possible initiator of PrP conversion^24^,^25^,^26^. To determine the change in thermal stability among different recPrP variants generated in this study, we used a CPM (7-diethylamino-3-(4-maleimidophenyl)-4-methylcoumarin) thermal stability assay^47^,^48^. Among all the recPrPs, recPrP^a^ had the highest melting temperature (T_m_ = 78.8 °C) (Fig. 7, Table 1). The T_m_ for recPrP^b^ was 74.1 °C, suggesting it is less thermally stable than recPrP^a^. All single amino acid variants except for recPrP^T191P^ had melting temperatures around 70 °C, and recPrP^T191A^ had the highest T_m_ among this group (70.3 °C), consistent with the idea that the α-helix prone alanine stabilizes the helical structure of recPrP. Interestingly, recPrP^T191P^ had the highest melting temperature (74.3 °C) among all recPrP variants, suggesting that this recPrP, with a shortened α-helix 2, is quite stable; this result is consistent with a recent report^34^. Importantly, there was no correlation between thermal stability and PrP conversion, either by PMCA or by amyloid fibril growth assay.

## Discussion

In this study, we investigated the influence of the string of four threonines on PrP conversion by using two *in vitro* PrP conversion assays. The PMCA assay showed that the transmission barrier between murine PrP^a^ and PrP^b^ can be recapitulated in recPrion propagation and that both residues 108 and 189 contribute to the barrier. The amyloid fibril growth assay revealed a strong influence of residue 189 on fibril growth, but residue 108 had little effect. Moreover, our results also showed a position effect in the threonine string. When Thr-191 was mutated to Val or Ala, the mutants converted to the infectious prion conformation with a reduced efficiency. But when Thr-191 was mutated to Pro, it could not be converted by PMCA, suggesting that this residue is likely to be part of the β-sheet core, but not the loop region of in the final infectious conformation. Finally, we have shown that the variations in thermal stability caused by these PrP polymorphisms or mutations do not correlate with the ability of these recPrP to convert to either amyloid fibrils or infectious prions.

The lack of correlation between PrP thermal stability and convertibility is not particularly surprising. The instability of PrP will lead to misfolding or aggregation, but infectious prion conformations and/or amyloid fibril structures are ordered protein aggregates with defined structures, which require the normal PrP not only to unfold, but also to gain the defined structure of infectious prions or amyloid fibrils. Since unfolded PrP does not spontaneously convert to ordered aggregates, simply increasing the instability of PrP will increase PrP misfolding, but not necessarily result in increased production of infectious prions or amyloid fibrils.

The PrP^a^/PrP^b^ transmission barrier was originally identified through genetic analyses of prion disease susceptibility in mouse strains^49^,^50^ and elegantly dissected by using knock-in mouse technology^20^,^21^. Here we used PMCA to show that the difference of two amino acids between recPrP^a^ and recPrP^b^ created a barrier to propagating recPrP^a^-adapted recPrion, and both residues contributed to this barrier. Note that by changing the PMCA parameters, it is possible to adapt the prion propagation to various types of prions, as illustrated by propagating mouse prion to hamster PrP^C^ via PMCA^51^. In order to study the PrP^a^/PrP^b^ transmission barrier, we did not make any adjustment and used PMCA parameters that propagate recPrion with recPrP^a^ to test all recPrP variants, which allowed us to detect differences in transmission efficiency. The compromised PMCA propagation with recPrP^b^ or the derivative mutants recPrP^L108F^ and recPrP^T189V^ was consistent with *in vivo* results^20^.

Relative to PMCA, the amyloid fibril growth assay is less restrictive, and all recPrP variants grew amyloid fibrils (Suppl. Fig. 1, Table 1). Similar to the report by Cortez *et al*.^44^, we found that recPrP^b^ was less efficient in forming amyloid fibrils. Unexpectedly, recPrP^L108F^ behaved similarly to recPrP^a^ in both unseeded and seeded reactions (Figs 3 and 5), suggesting that the properties of recPrP^b^ in amyloid fibril growth are largely determined by residue 189. This finding is consistent with observations from animal study^20^, but the lack of influence of residue 108 is not. Unlike PMCA products, none of the amyloid fibrils could infect cultured CAD5 cells (data not shown) or seed the PMCA reaction (Suppl. Fig. 2). These observations suggest that the amyloid fibril growth assay is a less faithful PrP-conversion assay than PMCA. Notably, it has been suggested that recPrP has the freedom to oligomerize to a large number of assemblies in the presence of 2 M GuHCl (a condition for growing PrP amyloid fibrils) and recPrP amyloid fibrils are able to adapt to the structure that is most suitable for the buffer conditions^44^,^52^. Thus, the misfolded recPrP has more freedom to adopt various structures under amyloid fibril growth condition, which may account for the less faithfulness by the amyloid fibril growth assay. Nonetheless, our finding that residue 189 is the major determinant of the fibril-growing property of recPrP^b^ reveals that the amyloid fibril growth assay does recapitulate certain aspects of *in vivo* PrP conversion.

**Figure 1:**
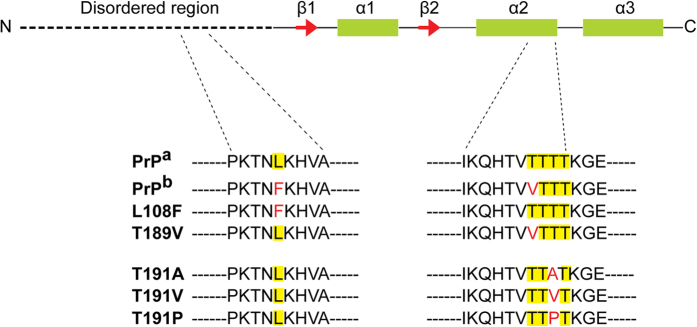
Schematic representation of murine PrP. PrP consists of a long N-terminal disordered region and a C-terminus that has three α-helices and two anti-parallel β-strands. The sequences of PrP^a^, PrP^b^, two derivative mutants (L108F and T189V), and three residue-191 mutants are shown; mutations are indicated in red.

**Figure 2:**
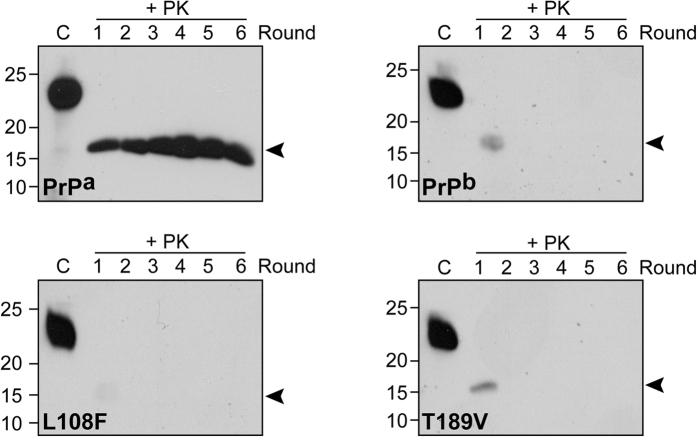
PMCA propagation of recPrion. Recombinant PrP^a^, PrP^b^, PrP^L108F^, or PrP^T189V^ was used as the substrate for PMCA. Each PMCA reaction was carried out for 6 rounds and PMCA products were digested with 50 μg/mL PK at 37 °C for 30 min. PrP was detected by immunoblot analysis with POM1 anti-PrP antibody^55^. “C” indicates undigested recPrP as a control. The original immunoblot images are in the Supplementary Figure S3.

**Figure 3:**
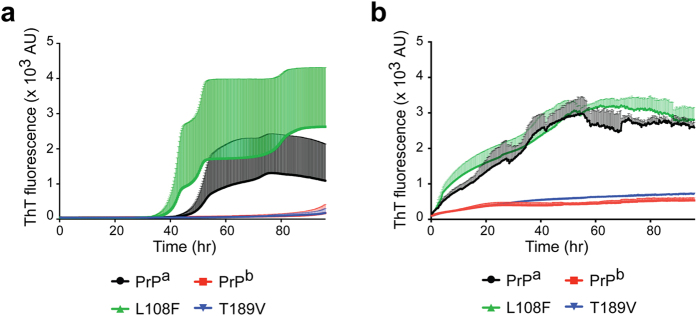
Amyloid fibril growth with recombinant PrP^a^, PrP^b^, PrP^L108F^, or PrP^T189V^. Thioflavin-T (ThT) kinetics of recombinant PrP^a^, PrP^b^, and two derivative mutants (L108F or T189V) in the absence (**a**) or in the presence (**b**) of seeds. The seeds used were pre-formed recPrP^a^ amyloid fibrils. Shaded areas are error bars, which indicate standard deviation.

**Figure 4:**
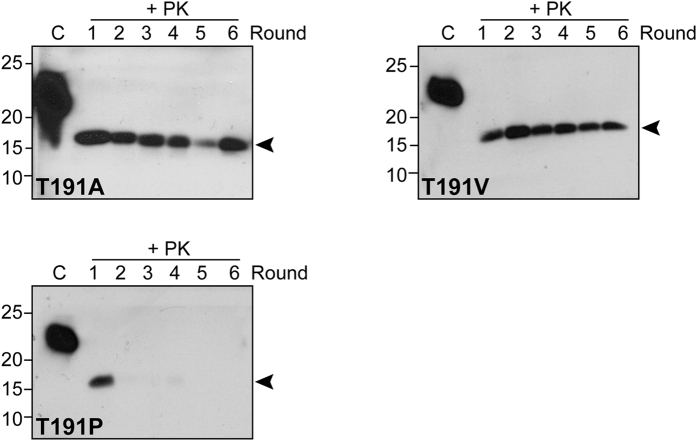
PMCA propagation of recPrion with recombinant PrP^T191A^, PrP^T191V^, or PrP^T191P^ as substrate. Each reaction was run for 6 rounds and PMCA products were digested with 50 μg/mL PK at 37 °C for 30 min. PrP was detected by immunoblot analysis with POM1 anti-PrP antibody. “C” indicates undigested recPrP as a control. The original immunoblot images are in the Supplementary Figure S3.

**Figure 5:**
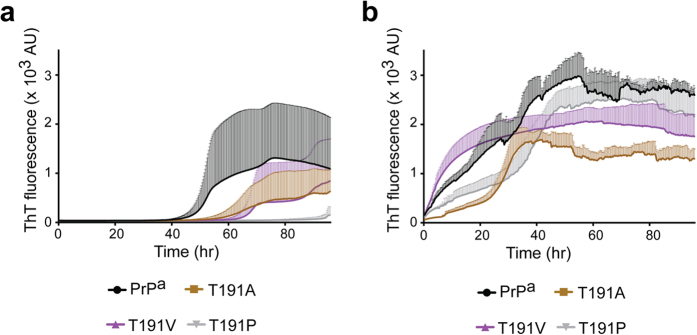
Amyloid fibril growth with recombinant PrP^T191A^, PrP^T191V^, or PrP^T191P^. ThT kinetics of recombinant PrP^a^, PrP^T191A^, PrP^T191V^, or PrP^T191P^ in the absence (**a**) or presence (**b**) of seeds (pre-formed recPrP^a^ amyloid fibrils). Shaded areas are error bars, which indicate standard deviation.

**Figure 6:**
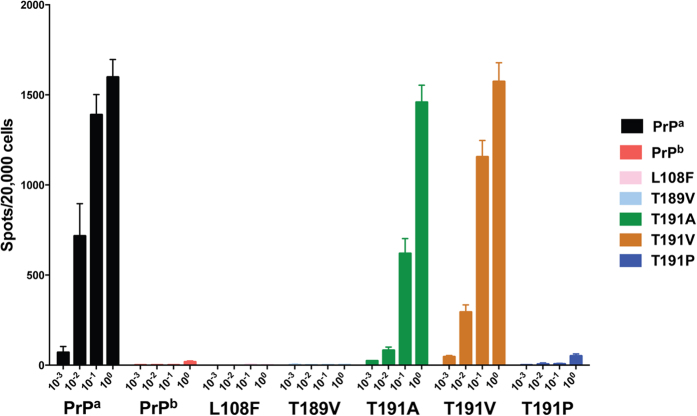
Elispot cell culture assay of prion infectivity. CAD5 cells were infected with serial 10-fold dilutions of each PMCA product as indicated. Elispot assay was performed by filtering 20,000 third-passage cells onto the membranes of Elispot plates, and the proportion of cells contained PK-resistant PrP was identified by enzyme-linked immunosorbent assay (ELISA).

**Figure 7:**
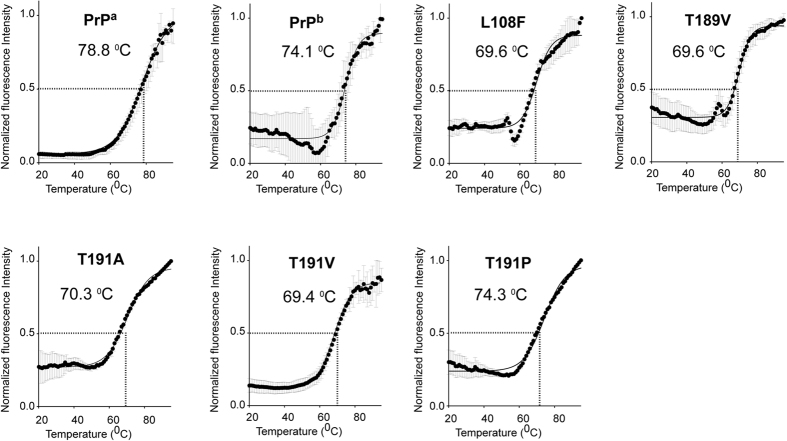
Thermal stability curves of recPrPs. The recPrP in deionized water (2 mg/mL) was used to obtain the thermal stability profiles. Melting temperatures (T_m_) were determined by fitting the curves to a Boltzmann sigmoidal equation using GraphPad Prism (version 6).

Thr-189 is at the beginning of the unusual four-threonine sequence in the α-helix 2 of PrP (Fig. 1), and the role of this string in PrP conversion is controversial^22^,^24^,^25^,^26^,^33^,^34^. In our PMCA study, all variants in this string had a reduced capability to form infectious prions, which could be due to the influence on three aspects of PrP conversion: (1) the seeding process, (2) the PrP conformational change, and (3) the final prion structure. Given that recPrion formed by recPrP^T191V^ or recPrP^T191A^ efficiently seeds wild-type PrP in CAD5 cells (Fig. 6), the amino acid mismatch between recPrP variants and wild-type PrP does not appear to significantly affect the seeding process. Because a single amino acid change is unlikely to alter the protein conformational change process, the most plausible explanation is that the threonine string forms part of the β-sheeted core of recPrion. This theory is consistent with our observation that replacing Thr-191 with Pro, a secondary structure breaker, strongly inhibits the propagation of recPrion by PMCA.

Although our data is consistent with the threonine string being a part of the β-sheeted core of recPrion, it does not suggest that this sequence is an obligatory part of the core in all prion strains. The finding by Munoz-Montesino *et al*. that deleting all four threonines does not affect the conversion of ovine PrP in RK13 cells^34^ indicates that this stretch is in the non-essential part in certain prion strains. Removing the threonine string may reduce the restraint of the final infectious prion structure of some prion strains, which would allow the PrP to better accommodate the incoming prion structure and propagate prion structures that it normally has difficulty propagating. This would explain the more efficient propagation of ovine PrP-adapted CJD strains by threonine deletion mutant^34^. Overall, our theory that the threonine string constitutes the β-sheeted core in certain prion strains, but is in the non-essential region in other strains explains the seemingly conflicting results, particularly the strong influence of residue 189 on prion transmission^20^ and the efficient prion formation by the threonine deletion mutant^34^.

It is important to note that the recPrion was formed *de novo* in a test tube with recPrP^36^, which might be different from native prions formed in animals. The PMCA and amyloid fibril growth are both *in vitro* assays, which may only capture certain aspects of the *in vivo* prion propagation process. Notwithstanding the limitations, these *in vitro* assays offer relatively pure systems allowing us to assess the direct effects of these amino acids on PrP conversion, which is difficult to achieve using *in vivo* systems. Moreover, the relevance of our findings to *in vivo* prion biology is supported by the facts that the recPrion causes *bona fida* prion disease in wild-type animals^36^,^38^. Further, our PMCA results are fully consistent, and our findings from amyloid fibril growth assay are partially consistent, with the results of *in vivo* prion transmission in knock-in mice^20^.

In summary, we compared the PMCA propagation of recPrion with the amyloid fibril growth assay; analyzed the complicated relationships among PrP thermal stability, PrP conversion, and prion infectivity, which have resulted in novel insights into the role of the threonine string in forming infectious prions. Confirmation that the threonine string can be part of the β-sheeted core or non-essential region in different prion strains may offer a novel view of the structural differences among various strains and help elucidate the molecular structure of infectious prions.

## Methods

### Site-directed mutagenesis

The mutant variants of the mouse PrP were generated from pPROEX HTb vector containing mouse PrP23–230 using the QuikChange site-directed mutagenesis kit (Stratagene). The primers are listed in the online supplementary information, which were synthesized by Integrated DNA Technologies, Inc. (IDT). All mutations were confirmed by DNA sequencing.

### Expression and purification of recPrPs

All recPrP proteins were expressed in *E. coli* BL21 (DE3) and purified according to a previously reported protocol^53^ with minor modifications. Briefly, inclusion bodies were collected (15,000 × *g*, 30 min, 4 °C), resuspended in 6 M Guanidine hydrochloride (GuHCl), 10 mM Tris-HCl, 100 mM Na-PO_4_ buffer, 10 mM β-mercaptoethanol (βME), pH 8.0, sonicated on ice until completely solubilized, and applied to a Ni-NTA column (Qiagen). The recPrP was refolded on the column by running a GuHCl gradient (from 6 M GuHCl, 10 mM Tris-HCl, 10 mM βME, pH 8.0 to 10 mM Tris-HCl, 100 mM Na-PO_4_ buffer, pH 8.0) at 1 mL/min. After wash, recPrP was eluted with 10 mM Tris-HCl, 100 mM Na-PO_4_ buffer, and 500 mM imidazole, pH 5.8. All eluted fractions were combined and dialyzed using 7,000 MWCO, SnakeSkinTM Dialysis Tubing (Thermo Scientific) for 1.5 h against 10 mM Na-PO_4_ buffer, pH 5.8, and then dialyzed overnight against deionized water. Protein samples were lyophilized (FreeZone 4.5, LABCONCO) and stored at −80 °C for further use.

### Protein misfolding cyclic amplification (PMCA)

The PMCA reaction was carried out as previously described^43^,^54^. Each PMCA round consisted of 48 cycles of sonication (0.5 min) and incubation (29.5 min), and 1/10 of the reaction mixture was transferred to a fresh substrate mixture to start a new round.

### Amyloid fibril formation assay

For amyloid fibril growth, 0.5 mg/mL of recPrP was incubated in 2 M GuHCl, 100 mM K-PO_4_ buffer, pH 6.5, and 20 μM thioflavin T (ThT). The reaction volume was 200 μL per well in 96-well plates (Corning, Lot No. 065514030). In the seeded reaction, seeding was achieved by adding 1 μL of mouse recPrP fibrils (0.5 mg/mL) to each well. The plate was incubated at 37 °C with continuous shaking on a mcrioplate reader (SYNERGY2, BioTek). The fibril kinetics was monitored by measuring ThT fluorescence intensity every 15 min using 440-nm excitation and 480-nm emission. The amyloid-formation kinetics was calculated from five replicates using GraphPad Prism (GraphPad Software, Inc.).

### The enzyme-linked immunospot (Elispot) cell infection assay

The Elispot cell infection assay was adapted from previous studies^45^,^46^ with minor modifications. Briefly, 200 μL of PMCA products at round 6 were collected and centrifuged at 100,000 × g, 4 °C for 1 hour and the pellets were washed twice, followed by centrifugation at 100,000 × g, 4 °C for 1 hour after each wash. After the final wash, the pellets were resuspended in 200 μL of CAD5 growth media (OPTI-MEM, 5% BGS and 1% penicillin and streptomycin) and sonicated for 30 seconds with 50% output (Misonic Sonicator XL2020). Each sample was serially diluted 10, 100, and 1,000 times and 60 μL of undiluted and diluted samples were used to infect CAD5 cells. After two 1:10 splits, 20,000 CAD5 cells/well were transferred to the Millipore 96-well Elispot plates (MSIPN4W) and subjected to the Elispot assay^45^,^46^. The images were taken by S6 Micro Analyzer (CTL Analyzers, LLC) and processed by the ImmunoSpot software (CTL Analyzers, LLC). The graph was generated using GraphPad Prism (GraphPad Software, Inc.).

### Thermal unfolding using CPM fluorescence dye

The thermal stability assay was performed as previously described^48^. Briefly, 4 mg of 7-diethylamino-3-(4-maleimidophenyl)-4-methylcoumarin (CPM) (Sigma) was dissolved in 1 mL of DMSO. The solution was diluted 20-fold with water to make a working solution, which was kept on ice during the experiment. Diluted dye (40 μL) was mixed with 480 μL of 2 mg/mL recPrP and used immediately while protected from light to reduce photobleaching. The thermal denaturation assay was performed in a total volume of 130 μL of reaction mixture, and the mixture was transferred to a sub-micro quartz fluorometer cuvette and heated at a rate of 2 °C/min in a Cary Eclipse fluorescence spectrophotometer (Agilent Technologies). The fluorescence signal was monitored as the temperature increased (excitation 387 nm, emission 463 nm). The assay was performed over the temperature range of 20–95 °C. Data were fitted using the GraphPad Prism program (GraphPad Software, Inc.), and the melting temperature (T_m_) was calculated from the Boltzmann sigmoidal equation. Each experiment’s calculations were based on an average of 4 replicates.

## Additional Information

**How to cite this article:** Abskharon, R. *et al*. The role of the unusual threonine string in the conversion of prion protein. *Sci. Rep.* **6**, 38877; doi: 10.1038/srep38877 (2016).

**Publisher's note:** Springer Nature remains neutral with regard to jurisdictional claims in published maps and institutional affiliations.

## Supplementary Material

Supplementary Information

## Figures and Tables

**Table 1:** Properties of rPrP variants.

Protein	PMCA Successful PMCA/total No. of PMCA	Amyloid fibril *De novo* growth	Amyloid fibril Seeded growth	T_m_ (˚C) (± SD)
PrP^a^	20/20	+++	+++	78.8 ± 3.5
PrP^b^	0/20	+	+	74.1 ± 2.2
L108F*	0/20	+++	+++	69.6 ± 2.1
T189V*	0/20	+	+	69.6 ± 1.6
T191A*	5/20	+++	+++	70.3 ± 1.1
T191V*	12/20	+++	+++	69.4 ± 2.2
T191P*	0/20	+	+++	74.3 ± 0.9

*The PrP variants were generated on rPrP^a^ background. Tm is the melting temperature of PrP variants. SD represents standard deviation.
