# Supplementary material for: The role of the unusual threonine string in the conversion of prion protein
Source: Sci Rep. 2016 Dec 16;6:38877. doi: 10.1038/srep38877 (PMC5159806; doi:10.1038/srep38877)
Supplement: Supplementary Information [file srep38877-s1.pdf]

SUPPLEMENTARY INFORMATION

The role of the unusual threonine string in the conversion of prion protein

Romany Abskharon[1,3], Fei Wang[1], Kayla J. Vander Stel[1], Kumar Sinniah[2], Jiyan Ma[1]*

[1] Center for Neurodegenerative Science, Van Andel Research Institute, Grand Rapids, MI 49503, U.S.A.

[2] Department of Chemistry and Biochemistry, Calvin College, Grand Rapids, MI 49546, U.S.A.

[3] National Institute of Oceanography and Fisheries (NIOF), 11516 Cairo, Egypt

* Jiyan.Ma@vai.org

Primers for generating recPrP variants

Mutant	Forward primer	Reverse primer
T189V	5' CAGCACACGGTCGTCACCACCACCAAGG 3'	5' CCTTGGTGGTGGTGACGACCGTGTGCTG 3'
L108F	5' CTTGGTGGCTACATGTTCGGGAGCGCCATG 3'	5' CATGGCGCTCCCGAACATGTAGCCACCAAG 3'
T191A	5' CACGGTCACCACCGCCACCAAGGGG 3'	5' CCCCTTGGTGGCGGTGGTGACCGTG 3'
T191V	5' CACGGTCACCACCGTCACCAAGGGG 3'	5' CCCCTTGGTGACGGTGGTGACCGTG 3'
T191P	5' CACGGTCACCACCCCCACCAAGGGGG 3'	5' CCCCCTTGGTGGGGGTGGTGACCGTG 3'

Atomic force microscopy imaging and substrate preparation

All atomic force microscopy (AFM) images were collected on a Multimode Nanoscope 8 AFM with a Nanoscope V controller and ScanAsyst mode using ScanAsyst Air tips from Bruker (Santa Barbara, CA). Images were obtained at a resolution of 512 x 512 pixels. Prior to imaging, the vertically engaging E-scanner was calibrated for accuracy in the *x, y, z* direction by using a 1-μm grid with a depth of 20-nm. Mica substrates were prepared for imaging as follows: 10 μL of the protein to be imaged was deposited on a freshly cleaved mica surface. After a 5-min incubation, the mica surface was rinsed with 200 μL of nanopure water and dried with compressed air. Images were corrected for tip artifacts and surface bowing only and were analyzed using Scanning Probe Image Processor (SPIP) software (version 6.5.2, Image Metrology A/S, Lyngby, Denmark).

Supplementary Figure S1

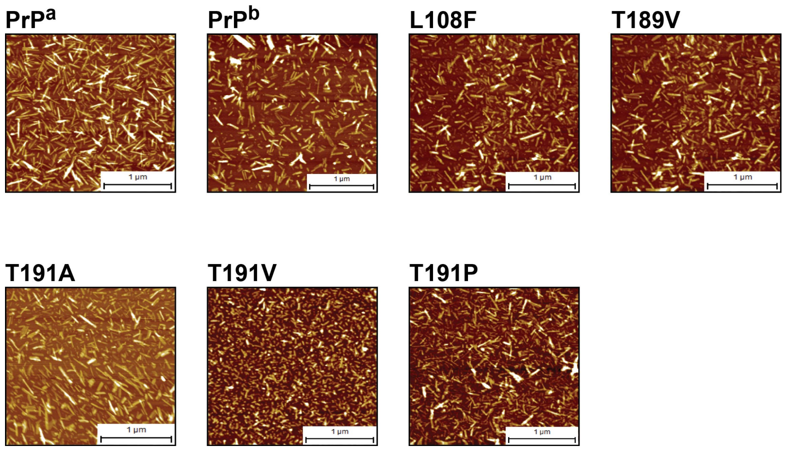

Figure S1: Atomic force microscopy topographical images of amyloid fibrils prepared with recPrPs as indicated. AFM imaging was performed at the end of the fibrillization reactions (60 h).

Supplementary Figure S2

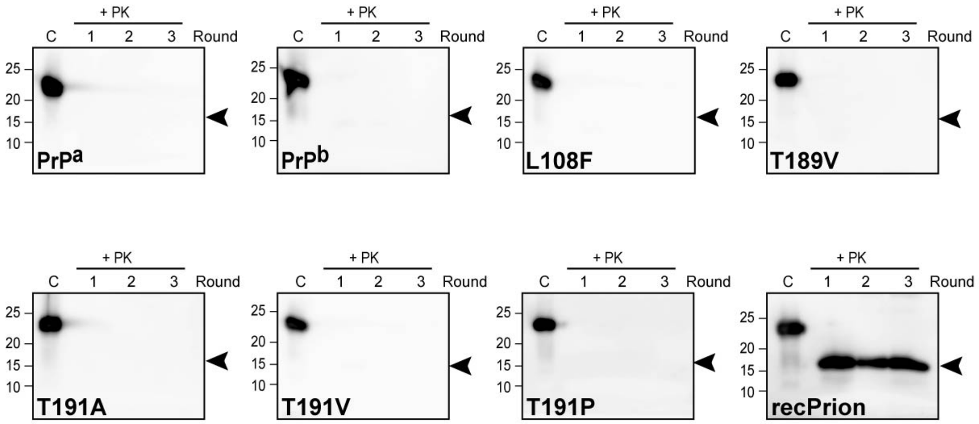

Figure S2: PMCA reactions were seeded with amyloid fibrils prepared with each recPrP variant as indicated. The recPrion was used as a positive control. PrP was detected by immunoblot analysis with POM1 anti-PrP antibody. “C” indicates undigested recPrP as a control.

Supplementary Figure S3

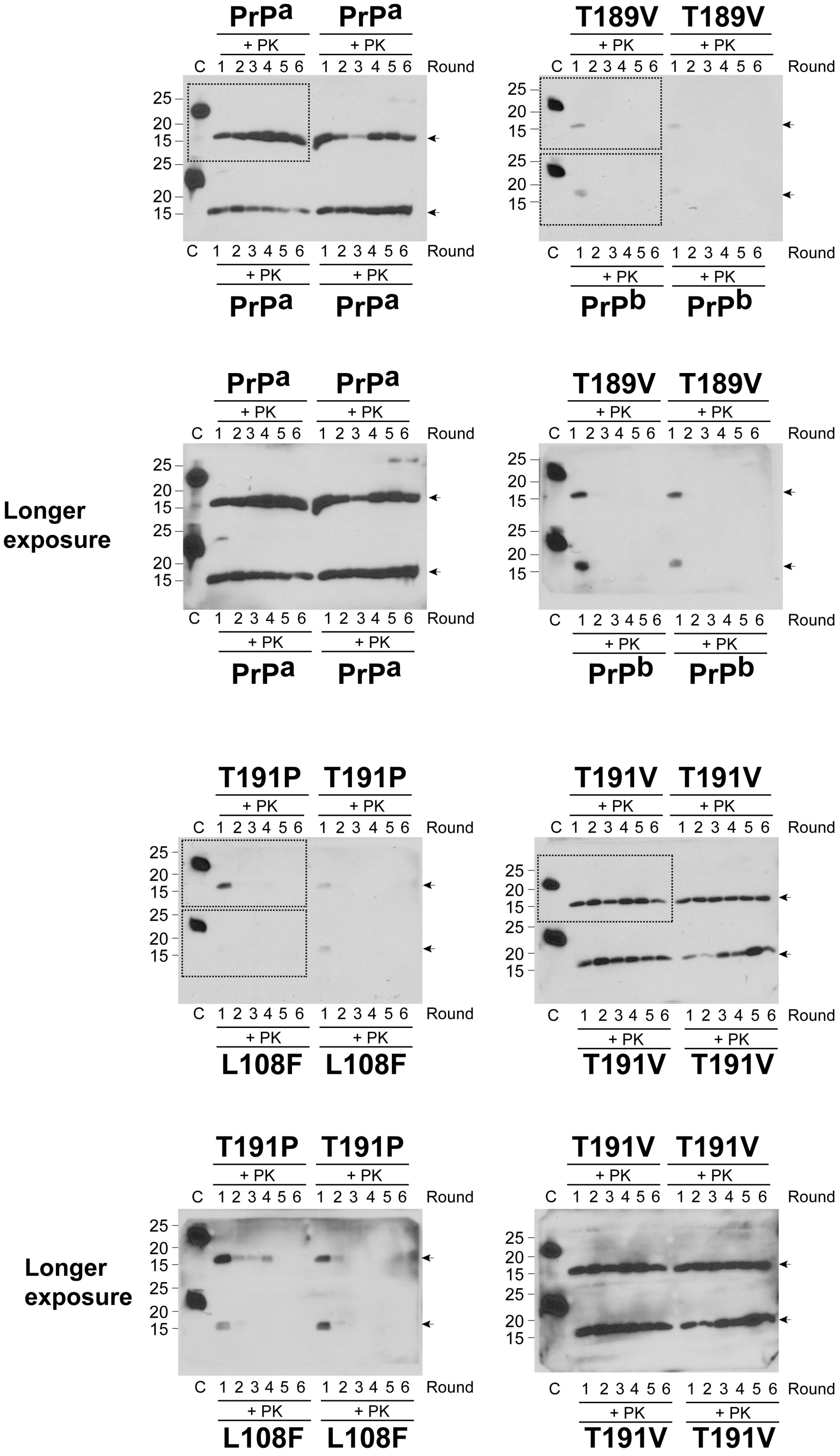

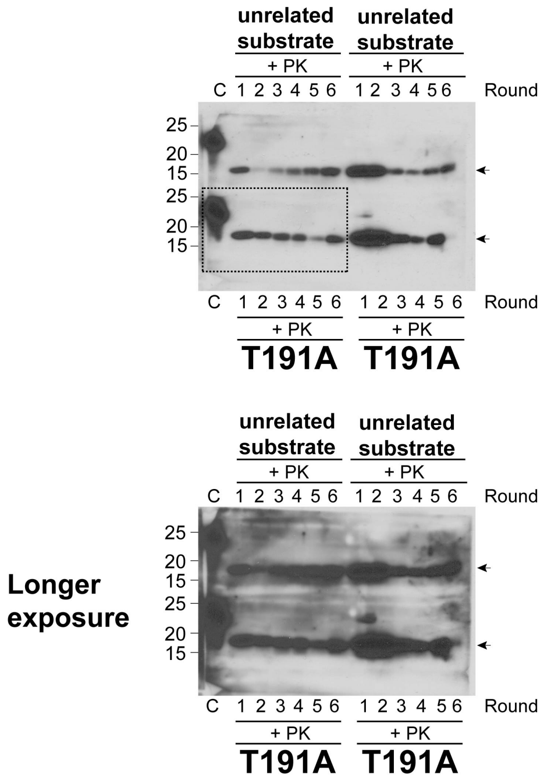

Figure S3: The original immunoblot images. Dotted boxes indicate the cropped images used in figures 2 and 4.
